# Study of Use of Dabigatran in Cerebral Venous Sinus Thrombosis

**DOI:** 10.7759/cureus.64744

**Published:** 2024-07-17

**Authors:** Satish Nirhale, Shalesh Rohatgi, Prajwal Rao, Pravin Naphade, Khushboo S Hatekar

**Affiliations:** 1 Neurology, Dr. D Y Patil Medical College, Hospital and Research Centre, Dr. D Y Patil Vidyapeeth (Deemed to be University), Pune, IND

**Keywords:** warfarin therapy, dabigatran etexilate, direct thrombin inhibitors, cerebral venous sinus thrombosis (cvst), therapeutic anticoagulation

## Abstract

Cerebral venous sinus thrombosis (CVST) is a challenging condition to diagnose and treat due to its diverse range of clinical presentations. The incidence of CVST is increasing, and although diagnostic techniques have improved, the mainstay of treatment is heparin followed by vitamin K antagonist (VKA), warfarin has remained largely unchanged for the past three decades. However, new direct oral anticoagulants (NOACs) like dabigatran have been developed to address the limitations of VKA therapy.

Magnetic resonance imaging (MRI) with magnetic resonance venography (MRV) is the current preferred diagnostic method for CVST due to its exceptional sensitivity and specificity.

This prospective observational study was set out to investigate the efficacy and safety of dabigatran in treating cerebral venous sinus thrombosis. The study included 30 patients who reported regular intake of 150 mg dabigatran etexilate twice a day. Among the participants, headache was the most commonly reported symptom.

The study found that patients treated with dabigatran experienced favorable outcomes, with all patients achieving re-canalization and reporting no major complications. These promising results suggest that dabigatran could be an effective treatment option for CVST cases. However, the study emphasizes the need for larger, multi-center studies to further validate these findings and improve the overall understanding of the condition and its treatment options.

## Introduction

Cerebral venous sinus thrombosis (CVST) makes up 10-20% of strokes in young individuals. The annual incidence ranging from 0.22 to 1.57 per 100,000. It's more common in women than men, with a female-to-male ratio of 3:1. Increased risk of CSVT is associated with pregnancy, puerperium, and oral contraceptives. The median age of patients with CSVT is 37 years, and only 8% of patients are older than 65. The risk of recurrent cerebral venous thrombosis (CVT) and venous thromboembolism after a first CSVT is low [1].

The most common initial symptom is a headache. Evaluation for an underlying pro-coagulant state may be rewarding for further prevention with long-term anticoagulation. In addition to this, symptomatic treatment of intracranial hypertension, seizures, visual disturbances and etiological treatment to manage the associated conditions is essential. CSVT can present in less common ways, including cavernous sinus syndrome, subarachnoid hemorrhage, and multiple cranial nerve palsies. There has also been a reported case of CVST mimicking a transient ischemic attack. Magnetic resonance imaging (MRI) of the brain with magnetic resonance venography (MRV) is currently the preferred diagnostic method due to its high sensitivity and specificity [2].

Antithrombotic treatment with heparin followed by oral anticoagulants is the standard therapeutic approach for CVST. New direct oral anticoagulants (NOACs) such as rivaroxaban, apixaban, edoxaban, and dabigatran, which are specifically aimed at factor Xa or thrombin, have been developed. Dabigatran etexilate is a prodrug of the direct thrombin inhibitor dabigatran which is a direct, reversible, potent inhibitor of thrombin. The major advantage of dabigatran is that the target is a single site in the coagulation cascade rather than multiple sites as vitamin K antagonists do, which makes it more potent for the prevention of hemorrhage. There are several studies elaborated in this article showing the non-inferiority of dabigatran in cerebral venous sinus thrombosis and grades of recanalisation. This study aimed to study the efficacy of direct oral anticoagulants (dabigatran) in the treatment of cerebral venous sinus thrombosis and adverse events like major haemorrhage with direct oral anticoagulants.

## Materials and methods

A prospective observational study was conducted at Dr. D. Y. Patil Medical College, Hospital and Research Centre, Pune, from May 2022 to May 2024. Ethics Committee of Dr. D.Y. Payil Vidyapeeth, Pune issued approval IESC/S.SP/02/2022. The present study was undertaken among 30 patients of CVST attending a tertiary care institute in western India, who were initially treated with clexane (weight-based dose) and dabigatran in a dose of 150mg twice a day. The dose of dabigatran was given as per the 2017 European Stroke Organization guidelines [3]. Results were assessed at the end of the six months of therapy while on dabigatran. A standard proforma for study participants was used for the collection of data. 

Inclusion criteria* *included patients aged more than 18 years and presence of clinical features suggestive of cerebral venous sinus thrombosis confirmed by neuroimaging findings diagnostic of CVST. Patients who have completed initial anticoagulation with a full dose of low molecular weight heparin followed by oral anti-coagulation with dabigatran in the dose of 150 mg two times a day for a duration of six months were included. Exclusion criteria included patients who have taken both warfarin and direct oral anticoagulants and patients aged less than 18 years of age.

Sample size and power calculation were based on preliminary data. A p-value < 0.05 was regarded as statistically significant (Power 90%, two-sided Type-I-error 5%). Multiple testing was corrected using Dunn’s correction (p=0.04, e=0.08, and z=1.96).

A modified Rankin score was used to assess the recovery and prognosis. Assessment of recanalization was done using the Qureshi grading system [3-9].

## Results

Age wise distribution was assessed. Out of 30 patients, 19 were males and 11 were females. Majority of the patients were males (63.3%), while 36.7% were females as mentioned in Table 1.

**Table 1:** Gender-wise distribution

Gender	Frequency	Percent
Male	19	63.3
Female	11	36.7
Total	30	100.0

As per the age distribution, the mean and median age of the patients were 35.5 years and 36.5 years, respectively as mentioned in Table 2.

**Table 2:** Age distribution SD: standard deviation, IQR: interquartile range

Age	Years
Mean	35.50
Median	36.50
SD	10.11
IQR	28,40.50

Overall, the most common symptom reported by our study participants was headache. Headache was present in 28 patients (93.3%) and absent in two patients (6.7) as mentioned in Table 3. Vomiting was present in four patients (13.3%) and absent in 26 patients (86.7%) as mentioned in Table 4. Seizure was present in five patients (16.7%) and absent in 25 patients (83.3%) as mentioned in Table 5. 

**Table 3:** Percentage of patients with headache

HEADACHE	Frequency	Percent
Yes	28	93.3
No	2	6.7
Total	30	100.0

**Table 4:** Percentage of patients with vomiting

VOMITING	Frequency	Percent
Yes	4	13.3
No	26	86.7
Total	30	100.0

**Table 5:** Percentage of patients with seizure

SEIZURE	Frequency	Percent
Yes	5	16.7
No	25	83.3
Total	30	100.0

Among the patients with headache, 22 patients (91.7%) had complete recanalisation and six patients had partial recanalisation. A comparison was made between patients with headache with partial and complete recanalisation. There was no significant association between headache and grade of recanalisation as mentioned in Table 6.

**Table 6:** Headache and association with grade of recanalisation P value <0.05= statistically significant

HEADACHE		GRADE OF RECANALISATION		p value[<0.05=statistically significant]
		Partial recanalisation	Complete recanalisation	
Yes	Frequency	6	22	1.000
	Percentage	100.0%	91.7%	
No	Frequency	0	2	
	Percentage	0.0%	8.3%	
Total	Frequency	6	24	
	Percentage	100.0%	100.0%	

Among the patients with vomiting, three patients (12.5%) had complete recanalisation and one patient had partial recanalisation (16.7%). A comparison was made between patients with vomiting with partial and complete recanalisation. There was no significant association between vomiting with grade of recanalisation as mentioned in Table 7. 

**Table 7:** Vomiting and association with grade of recanalisation P value <0.05= statistically significant

VOMITING		GRADE OF RECANALISATION		p value [<0.05=statistically significant]
		Partial recanalisation	Complete recanalisation	
Yes	Frequency	1	3	1.000
	Percentage	16.7%	12.5%	
No	Frequency	5	21	
	Percentage	83.3%	87.5%	
Total	Frequency	6	24	
	Percentage	100.0%	100.0%	

Among the patients with seizure four patients (16.7%) had complete recanalisation and one patient had partial recanalisation. A comparison was made between patients with seizure with partial and complete recanalisation. There was no significant association between seizure with grade of recanalisation as mentioned in Table 8.

**Table 8:** Seizure and association with grade of recanalisation P value <0.05 = statistically signifiacnt

SEIZURE		GRADE OF RECANALISATION		p value[<0.05=statistically significant]
		Partial recanalisation	Complete recanalisation	
Yes	Frequency	1	4	1.000
	Percentage	16.7%	16.7%	
No	Frequency	5	20	
	Percentage	83.3%	83.3%	
Total	Frequency	6	24	
	Percentage	100.0%	100.0%	

In the follow-up investigation after six months of treatment, complete recanalization and partial recanalization were noted among 24 patients (80%) and six patients (20%) who were positive for thrombus in the baseline as shown in Table 9.

**Table 9:** Overall recanalisation

OVERALL GRADE OF RECANALISATION	Frequency	Percent
Partial recanalisation	6	20.0
Complete recanalisation	24	80.0
Total	30	100.0

In the present study, mild bleeding was present in the four patients, which was recorded as the adverse outcome of the therapy, but no thrombotic events were recorded in majority of the patients (26 patients) as shown in Table 10.

**Table 10:** Complications

COMPLICATIONS	Frequency	Percent
Yes	4	13.3
No	26	86.7
Total	30	100.0

At the end of the six months, the overal grade of recanalisation was observed amongst all patients, six patients had grade I recanalisation, nine patients had grade II recanalisation and 15 patients had grade III recanalisation.

The majority of the patients had Grade III recanalization (50%), followed by Grade II (30%) and Grade I (20%) as mentioned in Table 11.

**Table 11:** Overall grade of recanalisation

OVERALL GRADE OF RECANALISATION	Frequency	Percent
Grade I	6	20.0
Grade II	9	30.0
Grade III	15	50.0
Total	30	100.0

The modified Rankin score (mRs) assessment at the end of six months showed that 24 patients had no disability, three patients had near complete and three patients had partial (mild disability).

As per the modified Rankin score, a majority (80%) had complete (no disability), while 10% reported near complete and mild disability as mentioned in Table 12.

**Table 12:** Modified Rankin score

MODIFIED RANKIN SCORE	Frequency	Percent
Complete	24	80.0
Near complete (no disability)	3	10.0
Partial (Mild disability)	3	10.0
Total	30	100.0

## Discussion

CVT must be treated quickly to avoid neurological damage or mortality caused by venous infarct and bleeding [3]. Over the past decade, direct oral anti-coagulants (DOACs) have been utilised for the treatment of CVT and offer several benefits over warfarin. These include more consistent pharmacokinetics, the elimination of the need for monitoring the international normalised ratio (INR) or adjusting the dose daily. Moreover, DOACs have shown comparable effectiveness for a reduced risk of intracranial hemorrhage when compared to warfarin [4].

Overall, the most common symptom reported by our study participants was headache (93.3%). This is in line with the findings of Ferro et al. in which 90% of their patients reported headache, and Wasay et al. reported the symptom among 93% of the patients [5,6]. Eighty percent of the patients had headaches in Mendoca et al.'s study [6]. While one patient (3.3%) reported diplopia and 6.7% had blurring of vision in our study, 10% and 11.7% of the patients in the Ferro et al. study had reported diplopia and loss of vision as symptoms, respectively [5]. In the present study, seizure was present in 16.7% of the patients. Similarly, Mendoca et al. reported 18.2% of patients reporting seizures [6]. Ferro et al. reported a seizure prevalence of 21.7%, and Wasay et al. reported it among 31% of their study participants [5,7]. The overall clinical pattern found in our study is in line with the symptoms reported among the CVST patients [8]. Radiologically, sagittal sinus was positive among 56.7% of our patients, while Ferro et al. reported a 45% involvement of superior sagittal sinus among their patients who were in the dabigatran group [5]. Wasay et al. reported that 57.8% of the patients in the NOAC group had superior sagittal sinus thrombus, which was in line with our findings [7]. In the current study, 23.3% of the patients showed right transverse sinus and 53.3% of the patients showed left transverse sinus involvement. While one patient (3.3%) had a deep venous sinus involvement in our study, a higher proportion of patients (13.3%) showed positive deep venous systems in the Ferro et al. study [5]. In our study, 26.7% of the patients had internal jugular vein involvement at the baseline, while Ferro et al. reported that 36.7% of their patients showed jugular vein involvement in the CVT [5]. Mendoca et al. reported a higher proportion of patients (46.7%) with jugular vein involvement [6]. Wasay et al. reported involvement of other sinuses, such as sigmoid, jugular vein, etc, among 64.4% of their NOAC patients [7]. At the end of the six months, the majority of the patients in our study had Grade III recanalisation (50%), followed by Grade II (30%). In contrast, Grade III collateral as well as recanalisation was reported among only one of their patients in the Farrag et al. study [8,9].

Overall, 80% of the CVST patients in the present study had complete re-canalisation, while 20% had partial re-canalisation, which shows improvement in all the patients who were administered the dabigatran oral therapy.

Ferro et al. reported that 40% of their patients showed no change in the thrombosed sinuses, while 60% had improvements [5]. Mendoca et al. reported a relatively low proportion of patients with full recanalisation (26.7%), and higher proportion of partial recanalisation (53.3%) than our study [6]. However, no recurrent thrombus was found in our study, which was also echoed by Ferro et al. [5]. Herweh et al. also reported complete recanalisation only among 57.6% of the patients [7,8].

In our study, after a six-month follow-up period, a modified Rankin score revealed that the majority (80%) had complete (no disability), while 10% reported near complete and mild disability, each. Ferro et al. reported a similar pattern, with 91.5% of patients having a score of 0-1 and 6.8% with a score of 2 [5]. Mendoca et al. reported that 86.7% of their patients showed an mRS of 0 to 1 score [6]. However, Wasay et al. reported a lower proportion of patients modified Rankin score of 0-1 among 65% of the patients, while 30% of the patients had a 2-3 score [7]. Farrag et al. reported that 17 of the patients followed up had improvements in terms of either recanalisation with or without collateral formation [9]. The process by which neurological function improves following CVST during the acute and post-acute phases is not clearly understood, but several mechanisms are suggested. These include the recovery from oedema, the clearing of hematoma, and the restoration of normal blood flow through recanalisation and/or the development of collateral vessels [9]. Lurkin et al. also reported a similarly favourable outcome in terms of mRS scores [10]. Thus, overall, dabigatran has shown better outcomes in terms of recanalisation and disability limitation among the CVST patients. In past studies, dabigatran is at least as effective as warfarin in conditions other than CVT and is associated with fewer bleeding incidents, particularly intracranial haemorrhages [11,12].

The present study is not without limitations. A small sample size limits the internal consistency of our findings. The lack of comparator groups (with other oral anti-coagulants and treatment modalities) limits the establishment of the comparative efficacy, superiority or equivalence of dabigatran to those treatment modalities. Since our study was undertaken among patients attending a single centre, the generalisability of our results to other settings is not possible.

## Conclusions

Dabigatran, a newer oral anticoagulant for treatment of patients with CVST, has yielded favourable outcomes, wherein all patients had achieved recanalisation (complete or partial). No major or serious complications were reported among the patients. Thus, dabigatran might be an effective drug for improving patient outcomes among CVST cases in the present settings. Further, multi-centric, analytical studies with adequate sample size where dabigatran is compared to other anti-coagulants needs to be undertaken to improve the internal and external validity of our study findings.
